# Meshed advancement flap for medium-sized vulvar defects after tumor excision

**DOI:** 10.1016/j.jpra.2026.06.016

**Published:** 2026-06-25

**Authors:** Ko Kagoyama, Teruhiko Makino, Shohei Kitayama, Keita Takemoto, Yu Matsui, Tadamichi Shimizu

**Affiliations:** Department of Dermatology, Graduate School of Medicine, University of Toyama, 2630 Sugitani, Toyama 930-0194, Japan

**Keywords:** Meshed advancement flap, Vulvar reconstruction, Extramammary paget disease, Squamous cell carcinoma, Staged reconstruction

## Abstract

Superficial vulvar defects of intermediate width are difficult to reconstruct when primary closure is not feasible and skin grafting or wider flap surgery may increase postoperative burden. We report 3 women who underwent staged reconstruction with a meshed advancement flap after tumor excision. Following temporary coverage with artificial dermis, reconstruction was performed 21–25 days later after confirmation of tumor-free margins. Defects measured 3 × 7, 3 × 5, and 4 × 10 cm. Operative time was 30–40 min, complete epithelialization occurred 10–14 days after reconstruction, and hospital stay for reconstruction was 5–10 days. No major complications or local recurrence during 5 years of follow-up were observed. This technique may offer a practical option for selected superficial vulvar defects of intermediate width.

Reconstruction of superficial vulvar defects of intermediate width remains challenging when direct closure is difficult and split-thickness skin grafting or wider flap surgery may increase donor-site morbidity and postoperative wound-care burden. Conventional vulvar reconstruction includes skin grafts and local flaps, but graft fixation in a moist field can be cumbersome and wider flap elevation may create additional wounds.^1^, ^2^, ^3^ A meshed advancement flap (MA flap), originally described for lower-leg defects, combines local advancement with multiple small tension-relieving incisions and may facilitate closure of wounds under tension.^4^^,^^5^ We describe our experience using this technique for staged reconstruction after vulvar tumor excision.

Three consecutive women underwent staged MA flap reconstruction at the University of Toyama between April 2019 and March 2021. Diagnoses were squamous cell carcinoma of the left labia minora in 1 patient and extramammary Paget disease of the left labia majora and of both labia majora/perineum in 2 patients. The MA flap was selected for superficial vulvar defects of intermediate width in which primary closure was not feasible, vaginal, urethral, or anal mucosal reconstruction was unnecessary, and bulk tissue replacement was not required. After tumor excision, wounds were temporarily covered with TERUDERMIS® Artificial Dermis (GC Corp., Tokyo, Japan). The artificial dermis was removed 2 weeks later in the outpatient setting. During this interval, after discharge, wounds were managed with gauze dressing changes without washing. Delayed reconstruction was performed 21, 22, and 25 days after excision, respectively, after histopathologic confirmation of tumor-free margins. During the week before reconstruction, patients washed the wound with soap, applied gentamicin ointment, and covered it with gauze. Written informed consent for publication of clinical details and images was obtained from all patients.

At reconstruction, several 1-cm slit incisions were placed 1 cm from the wound edge and 1 cm apart. After limited undermining with a thin layer of subcutaneous fat, the flap was advanced until closure was achieved under reduced tension. Defect sizes at reconstruction were 3 × 7 cm, 3 × 5 cm, and 4 × 10 cm. No vaginal, urethral, or anal mucosal reconstruction was required. Operative time was 30, 32, and 40 min, respectively. Complete epithelialization of the residual mesh openings occurred 10, 11, and 14 days after reconstruction. Hospital stay for tumor excision was 3, 3, and 10 days, whereas hospital stay for reconstruction was 7, 5, and 10 days; cumulative treatment-related stays were 10, 8, and 20 days. No wound dehiscence, infection, ischemia, or hematoma occurred. All surgical margins were negative, and all patients remained free of local recurrence during 5 years of follow-up. Clinicopathologic details are summarized in Table 1, and an intraoperative view from Case 3, which had the largest defect in this series, is shown in Fig. 1.

**Table 1:** Clinicopathologic and perioperative characteristics of the 3 patients.

Case	Age (years)	Diagnosis	Tumor site	Final margin status	Defect size at reconstruction (cm)	Interval from tumor excision to reconstruction (days)	Operative time (min)	Time to complete epithelialization (days)	Hospital stay for tumor excision (days)	Hospital stay for reconstruction (days)	Cumulative treatment-related hospital stay (days)	Postoperative complications	Follow-up duration (months)	Local recurrence
1	46	Squamous cell carcinoma	Left labia minora	Negative	3 × 7	21	30	10	3	7	10	None	60	None
2	76	Extramammary Paget disease	Left labia majora	Negative	3 × 5	22	32	11	3	5	8	None	60	None
3	47	Extramammary Paget disease	Both labia majora and perineum	Negative	4 × 10	25	40	14	10	10	20	None	60	None

Values are given per patient. Follow-up duration was calculated from reconstruction to the most recent clinical follow-up.

**Fig. 1:**
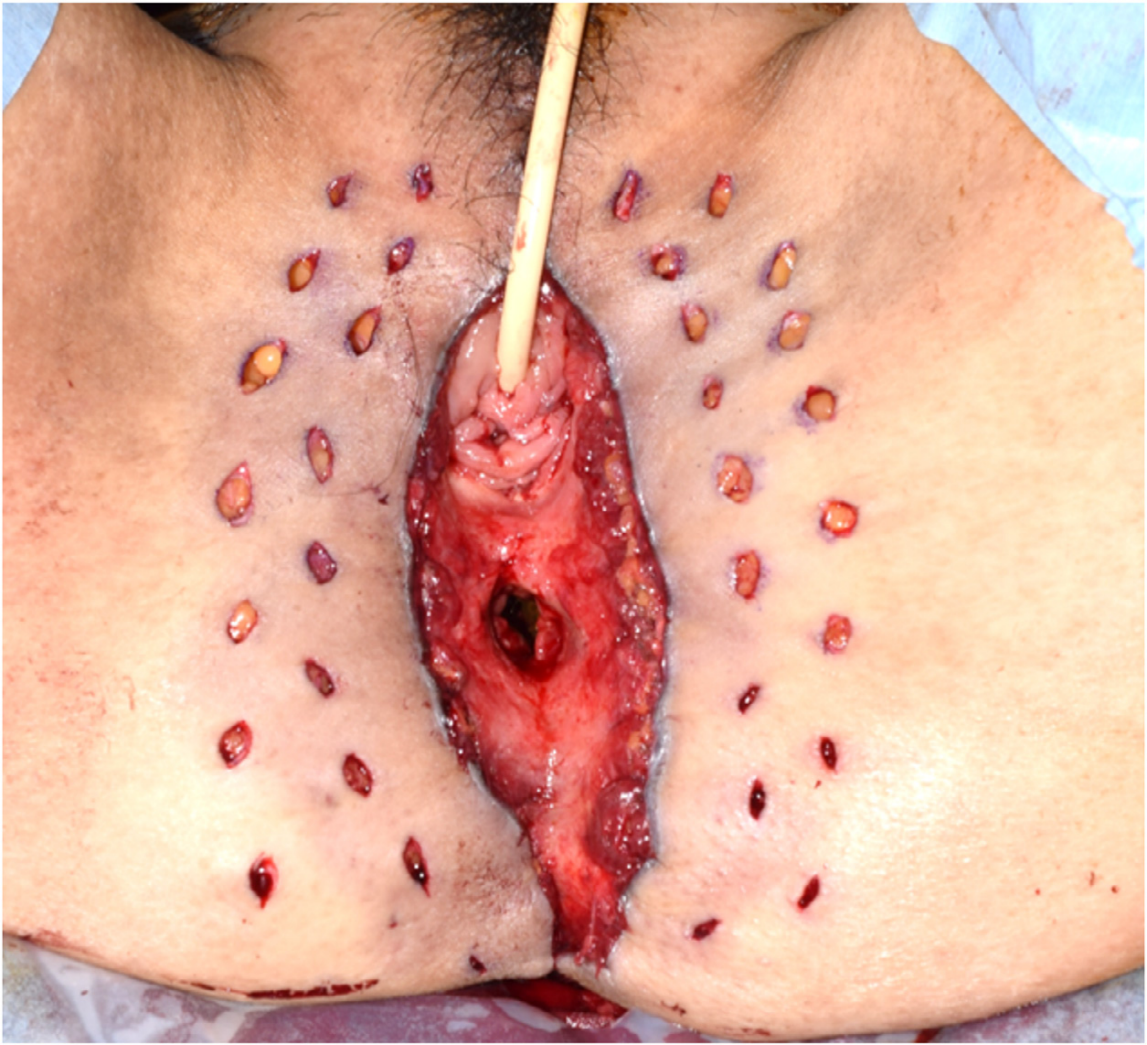
Intraoperative appearance of Case 3 during staged meshed advancement flap reconstruction, showing multiple 1-cm slit incisions placed adjacent to the defect to facilitate advancement and reduce closure tension.

This series suggests that the MA flap may be useful for selected superficial vulvar defects of intermediate width when direct closure is difficult but more extensive reconstruction is unnecessary. The technique was simple, with short operative times and discharge before complete epithelialization in all 3 patients. Although complete epithelialization required 10–14 days, the reconstruction admission was shorter, suggesting that postoperative management was feasible in the outpatient setting after discharge. Staged reconstruction also allowed confirmation of tumor-free margins before definitive closure, and granulation at the ulcer base during the interval may have reduced postoperative contour depression. The limitations of this report are the retrospective design, the very small number of patients, and the absence of a comparison group. Patient-reported outcomes were not formally assessed. Nevertheless, the MA flap may represent a practical option for vulvar defects of intermediate width after tumor excision, particularly when avoidance of graft fixation and wider flap dissection is desirable.

## Declaration of generative AI and AI-assisted technologies in the manuscript preparation process

During the preparation of this work the authors used ChatGPT (OpenAI) in order to improve language and readability. After using this tool, the authors reviewed and edited the content as needed and take full responsibility for the content of the published article.

## Funding

None.

## Ethical approval

Approved by the Medical Ethics Committee of the University of Toyama, Toyama, Japan (approval no R2026001).

## Declaration of competing interest

None declared.
